# Bioinspired caries preventive strategy via customizable pellicles of saliva-derived protein/peptide constructs

**DOI:** 10.1038/s41598-021-96622-y

**Published:** 2021-08-20

**Authors:** Dina G. Moussa, Walter L. Siqueira

**Affiliations:** College of Dentistry, University of Saskatchewan, Saskatoon, SK Canada

**Keywords:** Imaging, Peptides, Proteins, Translational research, Biofilms

## Abstract

Dental caries has been the most widespread chronic disease globally associated with significant health and financial burdens. Caries typically starts in the enamel, which is a unique tissue that cannot be healed or regrown; nonetheless, new preventive approaches have limitations and no effective care has developed yet. Since enamel is a non-renewable tissue, we believe that the intimate overlaying layer, the acquired enamel pellicle (AEP), plays a crucial lifetime protective role and could be employed to control bacterial adhesion and dental plaque succession. Based on our identified AEP whole proteome/peptidome, we investigated the bioinhibitory capacities of the native abundant proteins/peptides adsorbed in pellicle-mimicking conditions. Further, we designed novel hybrid constructs comprising antifouling and antimicrobial functional domains derived from statherin and histatin families, respectively, to attain synergistic preventive effects. Three novel constructs demonstrated significant multifaceted bio-inhibition compared to either the whole saliva and/or its native proteins/peptides via reducing biomass fouling and inducing biofilm dispersion beside triggering bacterial cell death. These data are valuable to bioengineer precision-guided enamel pellicles as an efficient and versatile prevention remedy. In conclusion, integrating complementary acting functional domains of salivary proteins/peptides is a novel translational approach to design multifunctional customizable enamel pellicles for caries prevention.

## Introduction

Dental caries is one of the most wide spread chronic diseases worldwide^1^. In addition, the treatment of dental caries consumes around 10% of the healthcare budget of industrial countries^2^. Caries typically originates in the enamel layer, then it progresses to involve deeper layers. Since the enamel is a unique tissue that cannot be healed or regrown^3^, dentists have no choice but proceeding with invasive treatments once the carious lesions are diagnosed after cavitation. Given the high rates of caries recurrence^4^, more invasive treatments may be required to the extent of root canal therapy or extraction. Nevertheless, new preventive approaches, excluding fluoride therapy, have been poorly studied and up-till the present time, we don’t have an efficient precision-guided preventive therapy.

Oral biofilm, key causative factor, starts by the acquired enamel pellicle (AEP) formation at the interface between the tooth and the oral environment^5^. AEP is a thin proteinaceous film derived from salivary proteins/peptides that are selectively adsorbed on enamel^6^. Once AEP forms, early colonizers adhere to it forming a foundation for biofilm succession^7^. Thus, modulating the AEP during its formation is critical for shaping the subsequent biofilm maturation. The proteome/peptidome of AEP has been of great interest as it consists of a number of naturally occurring salivary proteins/peptides that may provide bioinspired clues to develop potential enamel protective agents^8^. Recently, we analyzed the whole proteome/peptidome of the AEP where histatins and statherin were prevalent among the 130 identified proteins^9–11^.

Histatins have been known with their antimicrobial activity especially histatin 3 and 5 (Hist3) and (Hist5)^12^ while statherin might be said to inhibit bacterial adhesion, based on its observed inverse relationship with total supragingival biofilm^13^. Also, the phosphorylated serine residues in statherin family and histatin 1 (Hist1) increase the binding affinity to hydroxyapatite (HA) crystals promoting the resistance to proteolytic degradation^14^. Intriguingly, we identified novel salivary peptides that may retain or augment the functional properties of their native proteins since they are products of proteins after bacterial cleavage^15^. For example, we recognized the functional domain (DR9) located within the statherin N-terminal portion that exhibits a higher affinity for HA and significantly inhibits calcium phosphate precipitation^16^. Likewise, the functional domain (RR14) located within the middle portion of Hist3 that is responsible for its antifungal activity^17^. However, the specific biofunctions of many identified peptides as well as their hypothetical constructs remain unclear.

Therefore, this work is focused on the biofunctional characterization of selected prevalent AEP peptides to understand their role in cariogenesis. Furthermore, it provides a solid base for engineering stable synthetic peptides comprising multifunctional domains as customizable pellicle therapeutics to control biofilm growth. Our ultimate aim is to develop an effective, biocompatible and precision-guided prevention remedy for dental caries.

## Material and methods

### Ethics approval and human participants

All methods were carried out in accordance with relevant guidelines and regulations. All experimental protocols were approved by the Research Human Ethics Board of the University of Saskatchewan (Review number #1597). After an informed consent was obtained from all participants, stimulated whole saliva samples were collected from healthy, non-smoking adult male and female volunteers ranging from 25 to 39 years old. All participants weren’t taking any specific fluoride supplements, other than the normal community fluoridated water, and they did not exhibit signs of gingivitis/periodontal disease, active dental caries, or any other oral or systemic condition that could affect whole saliva composition. Whole saliva samples were collected 2 h after breakfast between 9:00 am to 11:00 am to minimize the circadian rhythm differences and then pooled to reduce the biological variation.

### Proteins/peptides synthesis

All protein/peptide candidates were synthesized (purity > 95%) by American Peptide (Sunnyvale, California, USA) and SynPeptide (Pudong, Shanghai, China). Their names, sequences and molecular properties are listed in (Table 1).

**Table 1:** Tested proteins/peptides and their molecular and bioinhibitory properties.

Proteins/peptides	Molecular and bioinhibitory Properties						
Name/sequence	No. of amino acids	Molecular weight (g/mol)	pI	Charge at pH = 5	Charge at pH = 7	GRAVY	MIC (µM)
**Statherin**							
DSpSpEEKFLRRIGRFGYGYGPYQP	43	5413.99	6.25	0.8	0	-1.29	> 512
VPEQPLYPQPYQPQYQQYTF							
**DR9**							
DSpSpEEKFLR	9	1304.42	4.68	-0.41	-1	-1.55	> 512
**DR9/2**							
DSSEEKFLR	9	1110.19	4.68	-0.67	-1.02	-1.54	> 512
**DR9-DR9**							
DSpSpEEKFLRDSpSpEEKFLR	18	2590.79	4.66	-0.82	-2.02	-1.55	> 512
**RR14**							
RKFHEKHHSHRGYR	14	1875.06	11	8.1	4.93	-2.67	384
**Histatin1**							
DSpHEKRHHGYRRKFHEKHHSHRE	38	4945.22	8.32	9	2.66	-2.09	> 512
FPFYGDYGSNYLYDN							
**Histatin3**							
DSHAKRHHGYKRKFHEKHHSHRG	32	4062.35	9.99	12.4	6.66	-2.3	128
YRSNYLYDN							
**Histatin5**							
DSHAKRHHGYKRKFHEKHHSHRGY	24	3036.33	10.2	12.2	6.6f.	-2.45	234

Molecular properties obtained from https://web.expasy.org/protparam/ and http://www.baamps.it/. The red amino acids in the sequences of proteins/peptides symbolize the phosphorylated serine residues. “pI” denotes the isoelectrical point; the pH at which the molecule carries no net electrical charge. GRAVY stands for grand average of hydropathy. The GRAVY value for protein/peptide is calculated as the sum of hydropathy values/hydrophobicity of all the amino acids, divided by the number of residues in the sequence. Minimum Inhibitory Concentrations (MICs) were determined against *Streptococcus mutans* U159 in micromolar (µM).

### Tested proteins/peptides groups

Total of 13 groups were tested divided to 6 native proteins/peptides, 6 designed proteins/peptides constructs, and whole saliva as a control. The native protein groups were (statherin, Hist1, Hist3, Hist5) and their functional domains (DR9 and RR14) derived from statherin and histatins respectively. The native groups represent the abundant proteins/peptides/fragments identified in the in vivo AEP^8,10^. The designed constructs were DR9/2, a unphosphorylated version of DR9, and the hybrid constructs (DR9-DR9, DR9 + RR14, DR9/2 + RR14, DR9/2 + Hist3, DR9/2 + Hist5) combining 2 functional domains derived from histatins and/or statherin families with or without being phosphorylated. The functional domains were either linked synthetically, such as DR9-DR9 or formulated as hybrid admixtures of 2 different domains, 50% each molarity-based, for the rest of the candidates. The control was a pooled whole saliva collected 2 h after meal in sterile 14-ml polypropylene tubes (Corning, NY, USA) chilled on ice. The collected saliva was spun down and filter-sterilized using 0.2 μm syringe filters.

### Minimum inhibitory concentration (MIC) assay

MICs were assessed for all proteins/peptides against *Streptococcus mutants* (*S. mutans*) U159 oral bacteria to identify the lowest concentration at which no bacterial growth is observed. Planktonic bacteria were grown aerobically (10% CO_2_) in brain heart infusion (BHI) medium (BD BBL™ #211,059, MD, USA) overnight. The proteins/peptides candidates were dissolved in 0.01% acetic acid (vehicle) and added to sterile 96-well polypropylene microtiter plates at decreasing twofold serial dilutions (0–512 μM). BHI medium was used as a control. Bacteria were inoculated to a final concentration of 3.0 × 10^6^ CFU/ml per well. The plates were incubated at 37 °C in a 10% CO_2_ atmosphere for 24 h under continuous shaking (200 rpm). Absorbance at 620 nm was measured using a microtiter plate reader (BioTek™ SynergyHT, VT, USA).

### Crystal violet (CV) biomass assay

CV assay was performed to assess the remaining bioburden on pellicle-coated substrates with protein/peptide test groups in comparison to human whole saliva. Microtiter plates and HA discs were used as substrates to be pellicle-coated before growing biofilms. Each substrate was pellicle-coated with 50 μl of 200 μM protein/peptide candidates for 2 h at 37 °C under gentle shaking. Pooled filtered whole saliva and the vehicle were used as positive and negative controls respectively. The unbound protein/peptide/saliva were removed, the adjusted inoculum was dispensed without or with 1% sucrose supplementation and the samples were incubated as mentioned above but without agitation^18^. Next day, the samples were rinsed and stained with 0.1% CV. After 15 min, the stain was rinsed, air dried, and photographed. All formed biofilms were structurally characterized with a bright field inverted microscope (EVOS™ xl core, MA, USA) before dissolving the satin, with 33% acetic acid, and reading at OD 620 nm.

### Live/dead vitality assay

The vitality of grown biofilms was observed alongside their biomass to determine the antimicrobial efficacy of protein/peptide pellicle-coatings against the attached bacteria in the biofilm state. All the steps are the same as mentioned above till the staining step. Working fluorescent solution was prepared by adding 3 μl of SYTO® 9 stain and 3 μl of Propidium iodide stain to 1 ml of filter-sterilized water. The staining solution was added to each sample very gently so as not to disturb the biofilm. After 20 min light-protected incubation, samples were rinsed with 0.9% NaCl and imaged with fluorescent inverted microscope (EVOS™ M5000, MA, USA) using 20 × lens. All assays were conducted 3 independent times with sample size n = 5.

## 2-Photon advanced bioimaging

### 3D renderings of biofilms grown on pellicle-coated HA discs

Z-stacks were acquired with the 2-photon microscope (Ultima IV, Prairie Technology Inc., WI, USA) for 3D structural and vitality characterization of biofilms grown on protein/peptide pellicle-coated HA discs. Non-descanned high sensitivity multi-alckali photocatode photomultiplier detectors (R3896, Hamamatsu, Japan) were used to allow visualization of multiple fluorophores located deep within the specimen. The infra-red (IR) laser (MAI TAI, Spectra-Physics, USA), tuned at 870 nm, was operated in pulsed mode using a 20 × NA1.0 water-immersion objective (XLUMPlanFLN, Olympus, Japan) with a 2 mm working distance suitable for deep imaging. Images were compiled and analyzed with FIJI software (Fiji Is Just) ImageJ, version: 2.1.0/1.53c https://imagej.net/Contributors^19^.

### 3D renderings of immobilization patterns of proteins/peptides on human enamel

This characterization method aimed to monitor the amount of protein/peptide molecules immobilized on enamel in pellicle-mimicking conditions to infer their binding capacity. Z-stacks for pellicle-coated enamel slices were acquired to monitor the immobilization patterns and thus, the binding capacity of fluorescently-labelled proteins/peptides. Enamel samples were ground to 0.5–0.6 mm and polished with 600 silica-grit papers using a polishing machine (Buehler, IL, USA). Samples were then ultrasonicated in a water bath for 20 min, dried and pellicle-coated with fluorescently labeled proteins/peptides as mentioned above. 2-photon images were acquired as aforementioned using 40 × NA 0.8 water immersion objective (LUMPlanFl/IR, Olympus, Japan). Solutions of the tagging molecule (FAM) and the vehicle, 0.01% acetic acid, were used to pellicle-coat the positive and negative controls respectively.

### Statistical analysis

Was performed using R, version 4.0.2.^20^ ANOVA and Tukey (HSD) post-hoc tests were applied for multiple comparisons. Differences were considered significant when p < 0.05.

## Results

**Figure 1:**
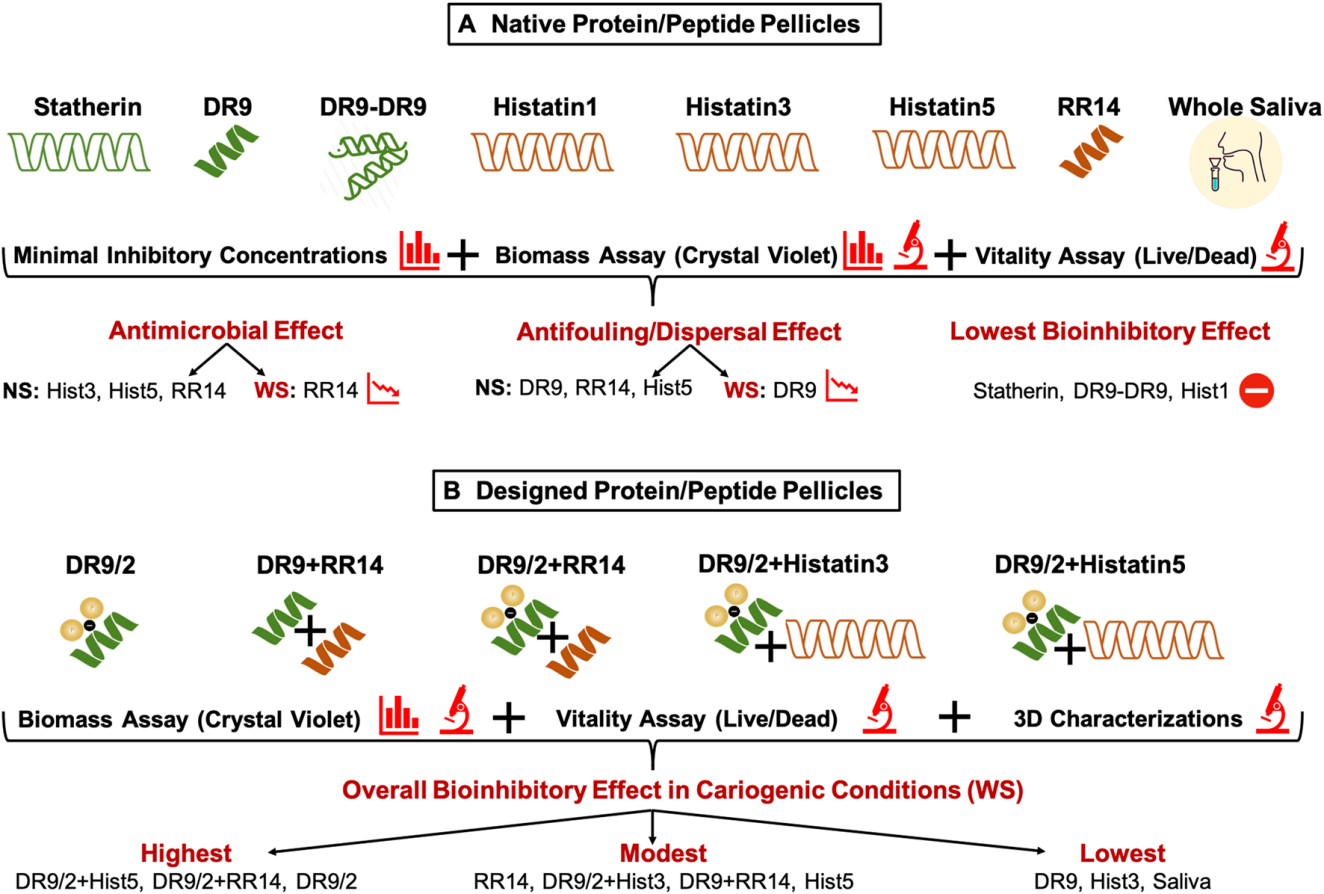
Schematic diagram depicting the workflow for studying the bioinhibitory effects of saliva-derived proteinaceous pellicles on the *S. mutans* U159 biofilms in normal and cariogenic conditions. (**A**) The native pellicles comprising the highly abundant proteins/peptides identified in the *in-vivo* human acquired enamel pellicles except for (DR9-DR9). The green color refers to the statherin protein family and its functional domain fragment (DR9) or the duplicated construct (DR9-DR9). The brown color refers to the histatins family and its functional domain fragment (RR14). All these candidates were tested for their bioinhibitory properties using qualitative (denoted by the microscope symbol) and quantitative (denoted by the bar graph symbol) assessment methods versus the human pooled whole saliva as a control in normal and cariogenic conditions. (No Sucrose “NS”) represents the normal conditions where biofilms were grown in brain heart infusion (BHI) medium without sucrose supplementation. (With Sucrose “WS”) represents the cariogenic conditions where biofilms were grown in BHI medium with 1% sucrose supplementation. Sucrose supplementation was included to assess the impact of disaccharides-mediated bacterial massive growth and associated high acidity on the behavior of tested candidates. The efficacy of all tested proteins/peptides/fragments was deteriorated in the challenging cariogenic condition; however, the significantly declined ones were specifically symbolized and the candidates showed the lowest bioinhibitory effects were excluded from further experimentation. (**B**) The novel pellicles were designed by either unphosphorylation of the DR9 functional domain, by removing the covalently linked phosphates at the 2^nd^ and 3^rd^ serine residues (DR9/2), or by hybridization of complementary-acting functional domains derived from the two different protein families. Our bioengineering strategies aimed to increase the biofilm dispersion and/or attain multifunctional synergistic effect in the challenging cariogenic (WS) conditions. All novel protein/peptide constructs were tested and characterized for their bioinhibitory properties in comparison to the native protein/peptide candidates in the sucrose-supplemented cariogenic condition. The overall bioinhibitory effect (the antimicrobial and/or the antifouling/biofilm-dispersal properties) were concluded in descending order in 3 categories (highest, modest, and lowest). Hist1, Histatin1; Hist3, Histatin3; Hist5, Histatin5.

We investigated the bioinhibitory effects comprising antimicrobial/antibiofilm, antifouling, and biofilm-dispersal capacities of the abundant native proteins/peptides versus our designed proteins/peptides constructs adsorbed at pellicle-mimicking conditions. The hybrid candidates comprise antifouling and antimicrobial functional domains derived from statherin and histatin families, respectively, to attain synergistic bioinhibitory effects (Fig. 1).

### Minimum inhibitory concentration (MIC) assay

Hist3 showed the strongest antimicrobial effect (MIC = 128 μM), followed by Hist5 (243 μM) and RR14 peptide (384 μM). All other proteins/peptides did not show any bacterial growth inhibition up to 512 μM (Table 1).

### Crystal violet (CV) biomass assay

**Figure 2:**
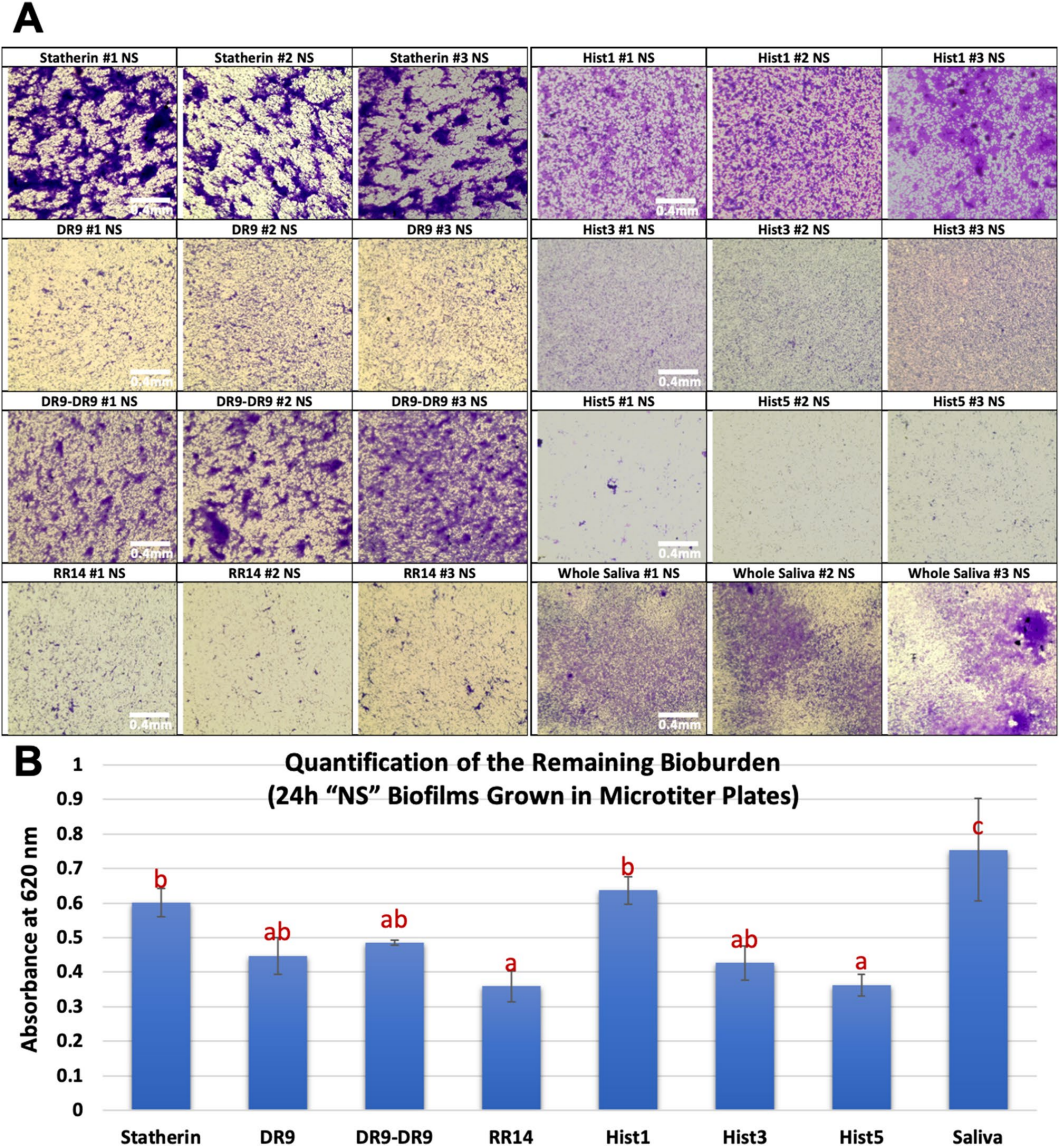
Crystal violet assay to assess the biomass of biofilms grown on native protein/peptide pellicle coatings: (**A**) Inverted brightfield microscope characterization of the crystal violet-stained 24 h *S. mutans* U159 biofilms. All biofilms were grown in polystyrene microtiter well plates pellicle-coated with native, except (DR9-DR9), protein/peptide candidates listed in Table 1. Filtered pooled whole saliva was used as a control. “NS” stands for “No Sucrose” where the biofilms were grown in BHI medium without sucrose supplementation. Scale bar is 0.4 mm. (**B**) Quantification of the remaining bioburden after dissolving in 33% acetic acid. Distinct lower-case letters show significant differences among groups (ANOVA and Tukey's (HSD) post-hoc tests, *p* < 0.05; Mean ± SD). Hist1, Histatin1; Hist3, Histatin3; Hist5, Histatin5.

**Figure 3:**
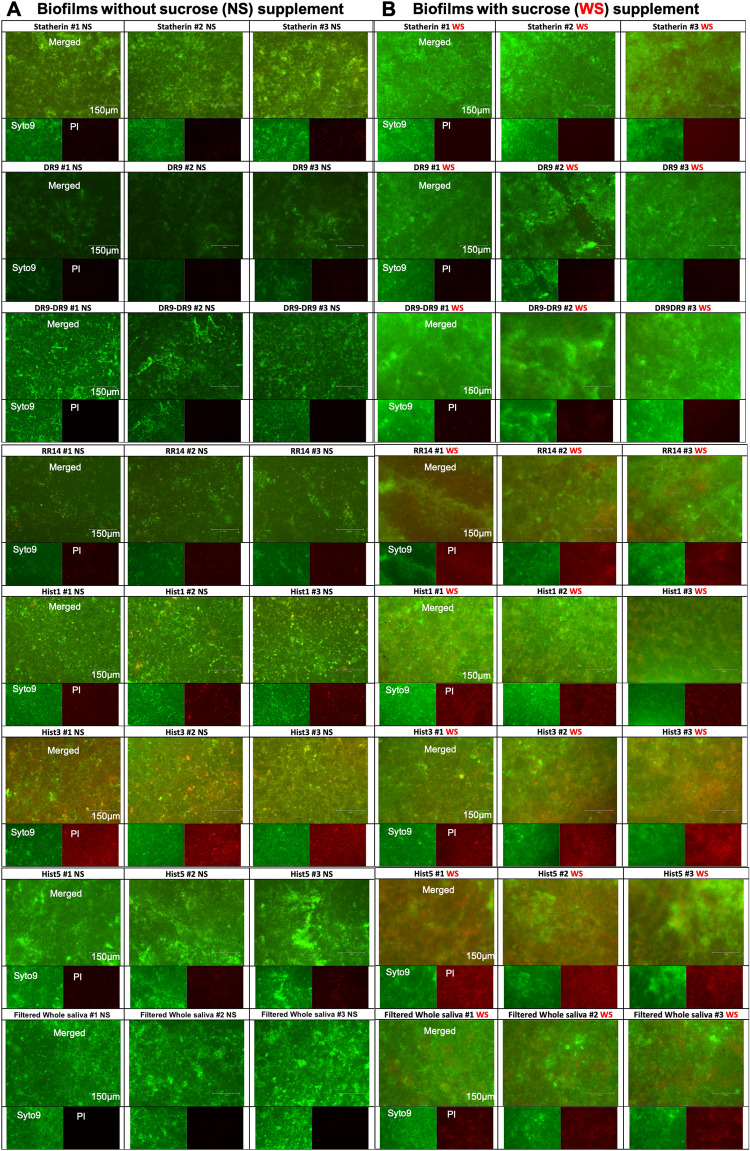
Live/Dead assay to assess the vitality of biofilms grown on hydroxyapatite (HA) discs pellicle-coated with native protein/peptide candidates under normal and cariogenic conditions: Inverted fluorescent microscope characterization of 24 h *S. mutans* U159 biofilms stained with Syto-9 “live/green” and Propidium-iodide “dead/red” stains. All biofilms were grown on HA discs pellicle-coated with native, except (DR9-DR9), protein/peptide candidates listed in Table 1. Filtered pooled whole saliva was used as a control. (**A**) “NS” stands for “No Sucrose” where biofilms were grown in BHI medium without sucrose supplementation. (**B**) “WS” stands for “With Sucrose” where biofilms were grown in BHI medium with 1% sucrose supplementation. Sucrose supplementation was included to assess the impact of disaccharides on the behavior of protein/peptide candidates. On each cluster, merged images are on the top and separated live channels are in the (bottom left) and dead channels are in the (bottom right). Scale bar is 150 μm. Hist1, Histatin1; Hist3, Histatin3; Hist5, Histatin5.

**Figure 4:**
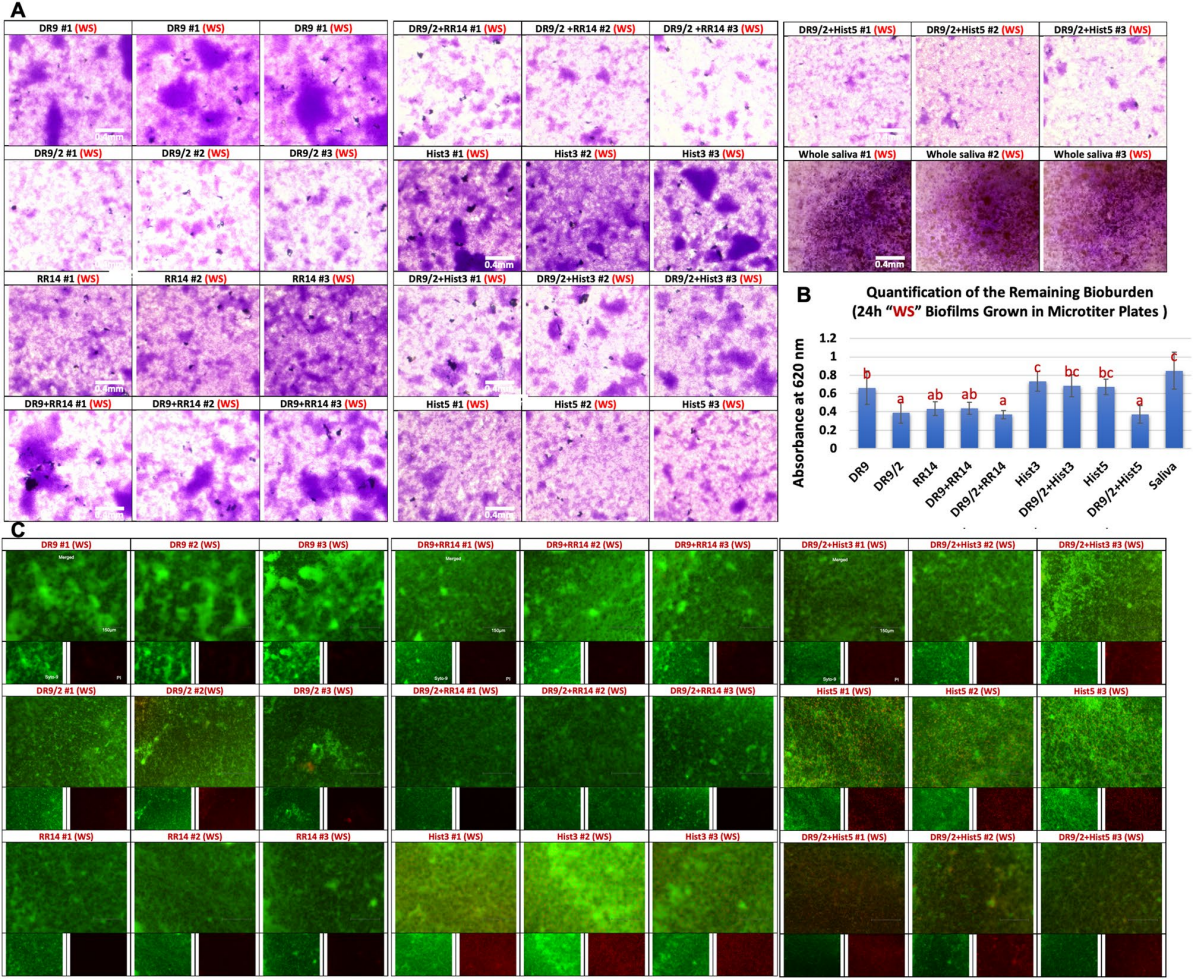
Biomass and vitality of biofilms grown in cariogenic conditions on pellicles of designed protein/peptide constructs: (**A**) Inverted brightfield microscope characterization of the crystal violet-stained 24 h *S. mutans* U159 biofilms. All biofilms were grown in microtiter plates pellicle-coated with designed novel protein/peptide constructs in comparison to native candidates. Filtered pooled whole saliva was used as a control. “WS” stands for “With Sucrose” where the biofilms were grown in BHI medium with 1% sucrose supplementation. Scale bar is 0.4 mm. (**B**) Quantification of the remaining bioburden after dissolving in 33% acetic acid. Distinct lower-case letters show significant differences among groups (ANOVA and Tukey's (HSD) post-hoc tests, *p* < 0.05; Mean ± SD). (**C**) Inverted fluorescent microscope characterization of 24 h *S. mutans* U159 biofilms stained with Syto-9 “live/green” and Propidium-iodide “dead/red” stains. On each cluster, merged images are on the top and separated live channels are in the (bottom left) and dead channels are in the (bottom right). Scale bar is 150 μm. Hist3, Histatin3; Hist5, Histatin5.

**Figure 5:**
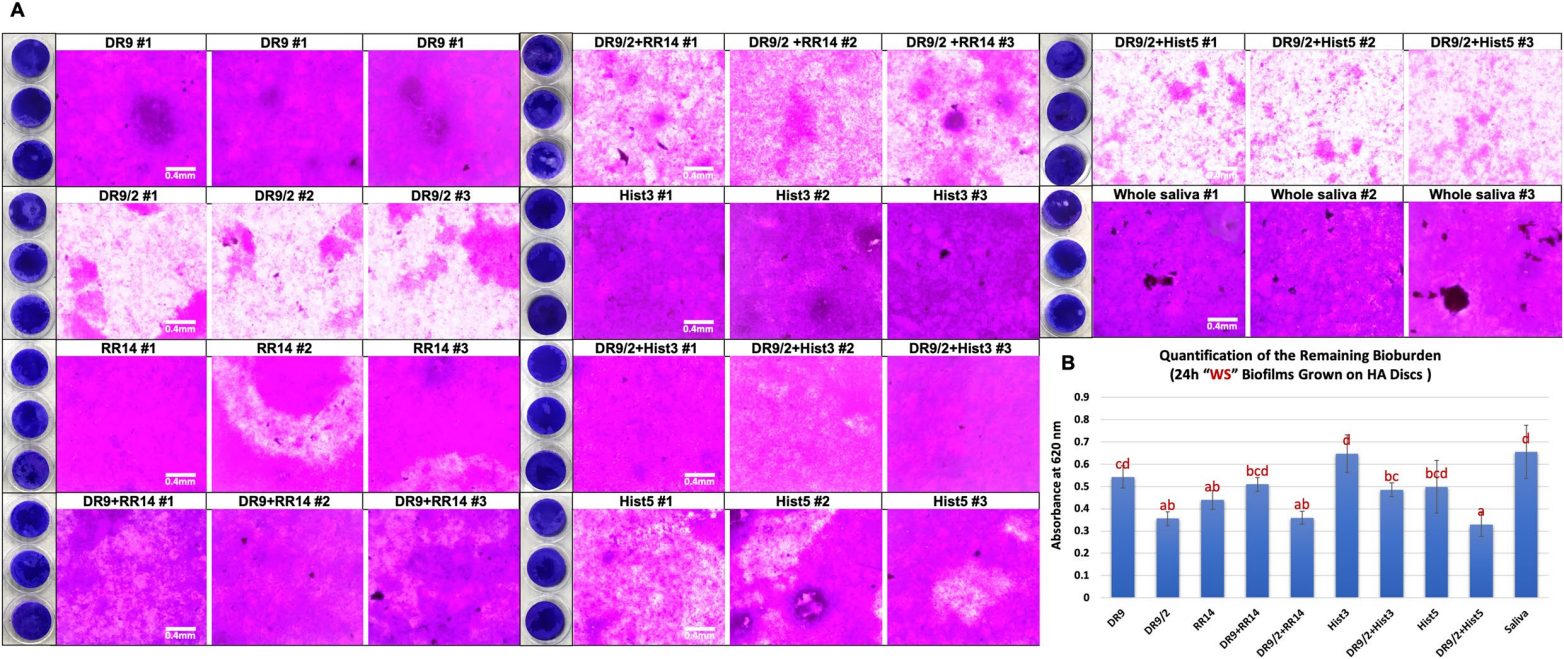
Biomass of biofilms grown in cariogenic conditions on hydroxyapatite (HA) discs pellicle-coated with designed protein/peptide constructs: (**A**) Inverted brightfield microscope characterization of the crystal violet-stained 24 h *S. mutans* U159 biofilms. All biofilms were grown on HA discs pellicle-coated with designed novel proteins/peptides constructs in comparison to native candidates. Filtered pooled whole saliva was used as a control. 3 representative HA discs are shown on the left of each group. Scale bar is 0.4 mm. (**B**) Quantification of the remaining bioburden after dissolving in 33% acetic acid. All biofilms were grown in BHI medium with 1% sucrose supplementation (With Sucrose “WS”). Distinct lower-case letters show significant differences among groups (ANOVA and Tukey's (HSD) post-hoc tests, *p* < 0.05; Mean ± SD). Hist3, Histatin3; Hist5, Histatin5.

The biomass was lesser in no sucrose “NS” condition than with sucrose “WS” cariogenic condition for all groups. In NS condition, Hist1, statherin, and DR9-DR9 showed the highest biovolume while RR14 and Hist5 recorded the least biovolume (Fig. 2A&B, 3). In WS condition, the novel/hybrid constructs, DR9/2, DR9/2 + RR14, and DR9/2 + Hist5 recorded the least biovolume (Fig. 4A, B). Structurally, biofilms associated with statherin family (statherin and DR9-DR9) characterized by clumps and aggregates (Fig. 2A) while more dispersed biofilms were associated with histatin family (Hist3, Hist5, and RR14) except Hist1 (Fig. 2A). With sucrose supplementation, DR9/2 and its hybrid constructs with RR14 or Hist5 showed significantly more biofilm dispersion compared to their counterparts on both substrates (polystyrene well plates and HA discs) (Figs. 4 and 5).

### Live/dead vitality assay.

Many dead cells were observed in Hist3 followed by Hist5, Hist1 and RR14 groups in both NS and WS conditions (Fig. 3). Statherin family didn’t show any dead cells in any condition; however, DR9/2 (WS) showed a notable biofilm dispersion compared to DR9 (Fig. 4C). For the hybrid constructs, integrating DR9/2 with Hist3 or Hist5 weakened the antimicrobial efficacy but increased the biofilm dispersion compared to Hist3 or Hist5 solely (Fig. 4C).

### 3D renderings of biofilms grown on pellicle-coated HA discs

**Figure 6:**
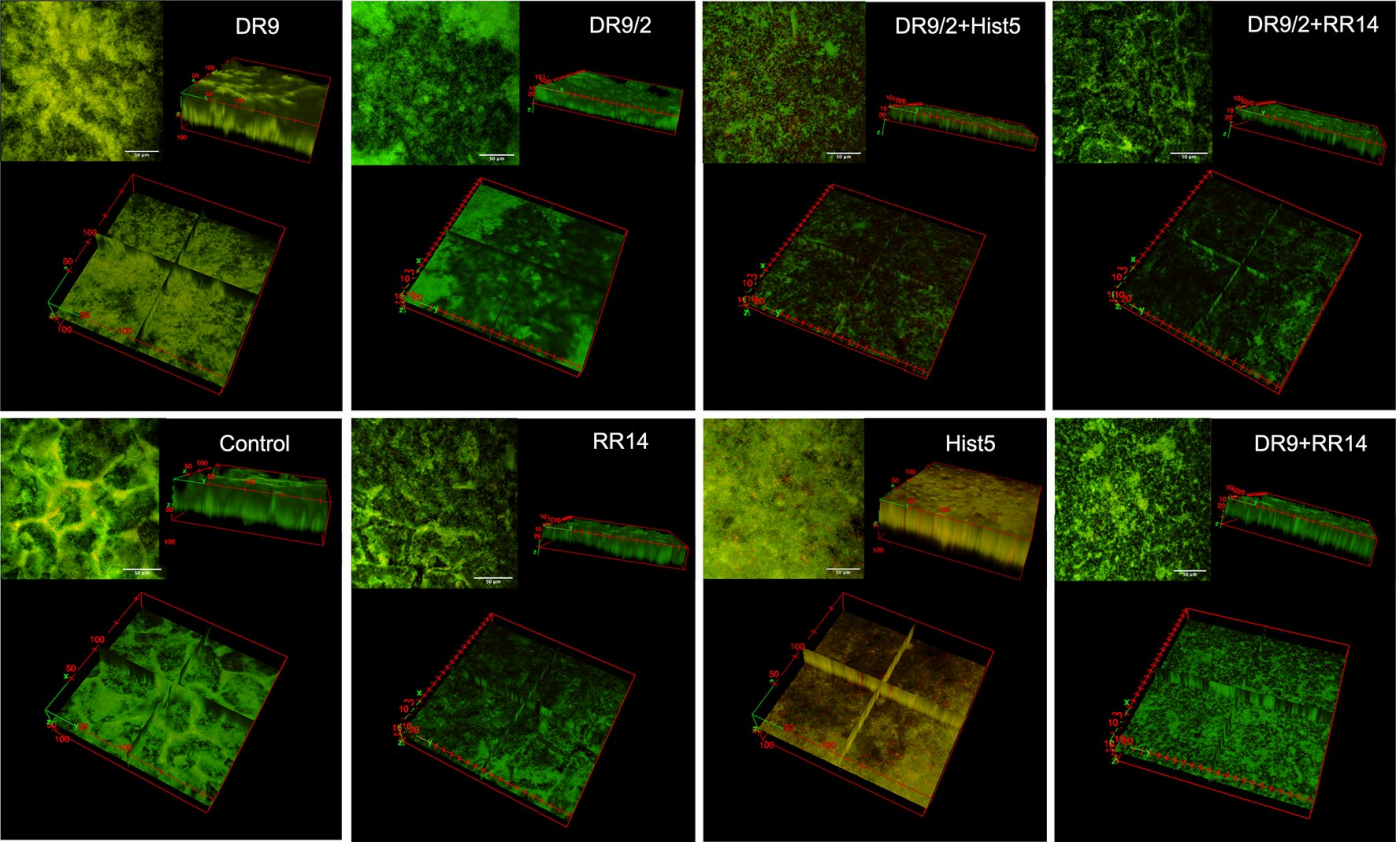
2-photon fluorescence 3D renderings of 24 h *S. mutans* U159 biofilms grown in cariogenic conditions on hydroxyapatite (HA) discs pellicle-coated with selected protein/peptide candidates: This characterization method aimed to monitor the biofilm structure and vitality across the entire biofilm thickness. Biofilms stained with Syto-9 “live/green” and propidium-iodide “dead/red” stains. On each cluster, 2D sum of z-stack slices “z-projection” is in the upper left (the scale bar is 50 μm), the 3D volume of biofilms is in the upper right, and an ortho middle slice of the z-stack is in the bottom. The z-projection is collapsing the data from 3 dimensions into 2 dimensions giving every pixel value an influence on the result. The 3D volume show the thickness of bacterial biofilms in μm. The ortho middle slice shows the biofilm’s structure and/or vitality at the middle of its thickness. Hist5, Histatin5.

Within the selected groups*,* DR/2 + Hist5 and DR9/2 + RR14 displayed the thinnest biofilms (~ 20 μm) followed by RR14, DR9/2 (~ 30 μm) and DR9 + RR14 (~ 35 μm). Controls and DR9 displayed the thickest biofilms (~ 60–65 μm) followed by Hist5 (~ 50 μm) (Fig. 6 and S2 Videos). The biofilm dispersion was highest in DR/2 + Hist5 group, this is in addition to the revealed non-vital cells, followed by DR9/2 + RR14, DR9/2, and RR14 domains (Fig. 6 and S1 Videos).

### 3D renderings of immobilization patterns of proteins/peptides on human enamel

**Figure 7:**
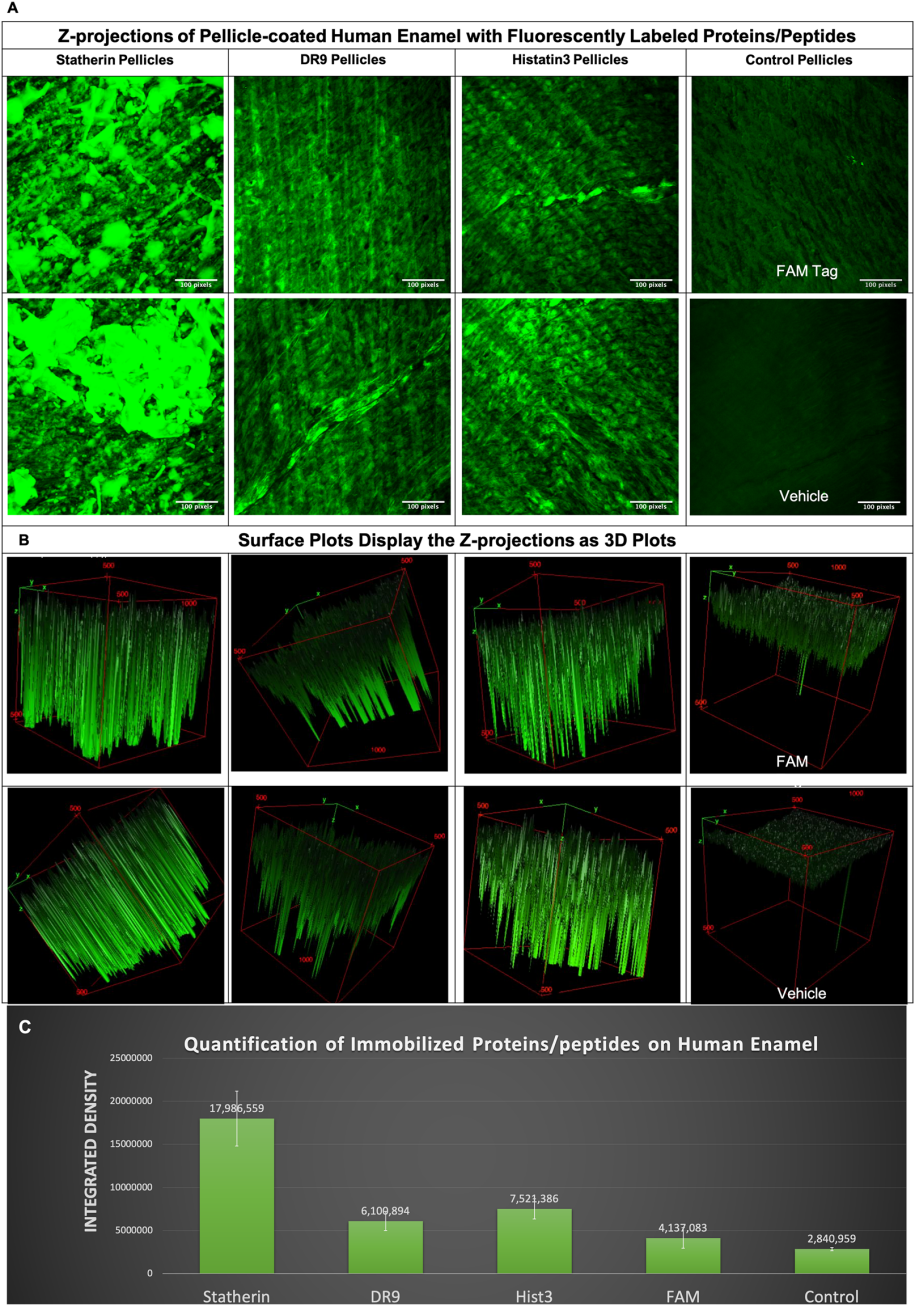
2-photon Z-projections and 3D surface plots of fluorescently labeled proteins/peptides immobilized on human enamel tissue: This characterization method aimed to visualize the immobilization pattern and binding capacity of representative proteins/peptides. (**A**) Maximum intensity z-projection showing the highest attenuation value throughout the volume onto a 2D image (the scale bar is 25 μm). (**B**) Surface plots display the images in panel A as 3D plots where the z-coordinate is representing the brightness of each pixel of the 2D image. FAM tag is the fluorescent molecule used to label proteins/peptides. The vehicle is 0.01% acetic acid used to prepare the solutions of tested proteins/peptides. (**C**) Quantification of the relative intensity “integrated density” of the immobilized proteins/peptides on human enamel tissue. Bars show average ± standard deviation. Hist3, Histatin3.

The amount of immobilized fluorescent protein/peptide molecules were quantified by calibrating the emitted fluorescence signals^21^^.^ The mean integrated density was calculated that sums all of the pixels along the acquired z-stacks, 4 images each. Statherin showed the highest immobilization capacity (the highest integrated density) followed by Hist 3 and DR9 (Fig. 7, S3 and S4 Videos). The FAM group showed the signals emitted from the tagging molecule itself where the vehicle group showed the autofluorescence emitted from the enamel tissue (Fig. 7, S3 and S4 Videos).

## Discussion

The caries process is initiated by dental plaque, encompasses diverse bacteria in the biofilm state, which is considered one of the most complex biofilm systems in nature^22^. Although different preventative approaches have been pursued for decades, none of them was efficient or precision-guided and therefore, caries remains the most common chronic disease globally^23^. Accordingly, there is a critical need for the exploration of new caries prevention strategies.

The AEP plays a fundamental lifespan protective role for the nonregenerative/nonrenewable enamel tissue^3,14,24,25^ that open clues for bioinspired approaches for caries reversion/prevention. Previous studies have recognized the vital role of the AEP in dental homeostasis given its nature as a selectively permeable membrane and its capability to neutralize acidic bacterial byproducts^6,11,25,26^. Additionally, it controls the composition of early colonizers and consequently, the successive layers of the microbial biofilm and the eventual dental plaque^27–29^. Despite of, very little is known about the nature of the different interactions between the AEP’s proteins/peptides and oral bacteria.

In our studies of the AEP proteome/peptidome, several functional protein domains were recognized which are the products of proteins after bacterial cleavage^9,10^. These domains typically intensify the functional properties of their native proteins and are more resistant to degradation^8,14,30^. Correspondingly, studying the AEP components and their functions has gained much attention as reviewed in^31^. Moreover, duplication/hybridization of selected synergically-acting domains have been proposed aiming to develop synthetic peptides with synergistic influence as therapeutics against dental caries and periodontal diseases^6,32–34^. Our group has previously studied the effects of targeted duplication/hybridization on hydroxyapatite crystal growth, controlling enamel demineralization, and resisting oral fungal activity^16,35,36^.

In this work, we sought to comprehensively-study the antimicrobial, antifouling, and biofilm dispersal properties of certain prevalent AEP proteins/peptides as well as proposing novel competent hybrid constructs (Fig. 1). The ultimate aim is to identify the key players that mediate biofilm inhibition/dispersion to engineer bioinspired multifunctional pellicles for caries prevention. We chose *S. mutans* UA159 because this strain is naturally competent and contains all of the genes essential for competence and quorum sensing^37^. This is beside the fact that it is the most cariogenic of all oral *streptococci* and capable to metabolize a wide variety of carbohydrates than any other Gram-positive organism^38^.

Initially, we screened the antimicrobial efficacy of the solution form of all tested protein/peptide candidates against *S. mutans* U159 in the planktonic state where we found Hist3 possesses the highest antimicrobial efficacy followed by Hist5 then the functional domain RR14. Conversely, the statherin and its fragments (DR9, DR9/2, DR9-DR9) didn’t show any bacterial growth inhibition. Since the AEPs are always in the adsorbed state and the overlaid biofilms are way more challenging than suspension cultures, we sought to assess the antimicrobial impact of tested proteins/peptides, immobilized in a pellicle-mimicking state, on growing biofilms. Our pellicles were developed by incubating the tested substrate, microtiter plates and/or HA discs, coated with the protein/peptide candidates for 2 h at the physiological body temperature to simulate the native AEP progression that typically reaches a plateau after 2 h^6^ . After growing biofilms, we observed a considerably reduced biomass build-up in pellicle-coated samples with RR14 and Hist5 followed by DR9 and Hist3 compared to whole saliva. Contrariwise had been noticed for statherin and Hist1 (Fig. 2). Interestingly, DR9 showed a considerable antifouling activity leaving a sparse biofilm architecture (Fig. 2A) and reduced biovolume (Fig. 2B) although it didn’t show any bacterial growth inhibition (Table 1). Likewise, RR14 displayed an antifouling efficacy that is comparable to Hist5 and superior to its parent (Hist3) (Fig. 2) despite of its modest MIC value (Table1). Accordingly, it is discernible that the specificity of bacterial attachment to the different proteins/peptides pellicles varies^28^ and antimicrobial and/or antifouling influences of the tested candidates are not necessary correlated. Noteworthy, cationicity and hydropathy are essential features that determine the selectivity and potency of proteins/peptides toward the anionic, negatively charged, phospholipids of bacterial membranes^39,40^. That might explain the antimicrobial behavior of the positively charged and less hydrophobic histatin family compared to the negatively charged and relatively more hydrophobic statherin family (Table 1 and Figs. 2, 3). In another aspect, we noticed that the biofilm architecture differs among the tested groups regardless of the biomass volume, where some biofilms were highly dispersed compared to others that have clumps and aggregates. Generally, we observed that biofilms associated with histatin family showed a notable dispersion compared to statherin family (Fig. 2A). Within each family, we spotted that the less phosphorylated serine residues, the more biofilm dispersal. Specifically, biofilms grown on Hist1 pellicles have several lumps compared to Hist3, Hist5, and RR14, given that Hist1 has a second phosphorylated serine residue where the other candidates are unphosphorylated (Fig. 2A). Similar wise for statherin family, where statherin (phosphorylated at the second and third serine residues) associated biofilms are less dispersed compared to its singly phosphorylated fragment DR9 (Fig. 2A).

Regarding the vitality of biofilms, no dead “red” bacterial cells could be visualized accompanying statherin family; though, the scattered vital “green” cells confirmed the antifouling behavior of DR9 in absence of sucrose (Fig. 3). In contrast, abundant dead cells were visualized throughout the grown biofilms of Hist3 group (Fig. 3). The sucrose supplementation was included to study whether the presence of this disaccharide would abrogate the observed bioinhibitory effects of tested candidates in the low-pH cariogenic environment. In sucrose condition, neither Hist3 nor DR9 maintained the low bioburden unlike RR14 and Hist5 that seemed to relatively hold their bioinhibitory capacity in such a bacterial growth-promoting environment (Figs. 3, 4).

Based on our observation that the more phosphorylated pellicle, the less dispersion of overlying biofilm, we introduced the unphosphorylated version of the antifouling DR9, a peptide DR9/2 to our test groups. Additionally, we integrated DR9/2 with the most antimicrobial tested proteins/peptides (Hist3, Hist5, RR14) as a hybrid solution to design potential multifunctional candidates. Also, we narrowed our focus to the sucrose-supplemented cariogenic condition and excluded the candidates with the least bioinhibitory efficacy (statherin, DR9-DR9, and Hist1) from further experimentation.

Promisingly, DR9/2 demonstrated a consistent dispersion and reduction of biofilms compared to its phosphorylated version on microtiter plates and HA discs (Figs. 4, 5). DR9/2 + RR14, DR9/2 + Hist5 constructs also showed comparable reduced biomass volume and evident biofilm dispersion (Fig, 3, 4). To better visualize the biofilm thickness and vitality across the grown biofilms on promising pellicle constructs, we implemented the 2-photon advanced microscopy to enable deep bioimaging^41^ that is inaccessible to the conventional 1-photon confocal microscopy^42^. The middle slices of z-stacks clearly revealed the dispersion behavior of RR14 as well as DR9/2 either solely or in hybrid solutions compared to DR9 in same conditions (Fig. 6 and S1 Videos). 3D renderings of whole biofilms evidenced that unphosphorylated DR9 substantially reduced biofouling by half with respect to its phosphorylated version. Likewise for the reduced biomass of the integrated DR9/2 with Hist5 group compared to Hist5 alone regardless of the triggered bacterial cell death (Fig. 6 and S2 Videos). Further tuning of different concentrations/ratios of the promising functional domains within the constructs is still under study to attain the maximum synergism.

Utilizing our expertise of implementing the 2-photon bioimaging on dental hard tissues^39,41^, we monitored the 3D immobilization capacity of representative fluorescently-labelled protein/peptide candidates on human enamel.

The data revealed that the doubly phosphorylated statherin showed more immobilized molecules, highest relative signal intensity, compared to the singly phosphorylated DR9 or the unphosphorylated Hist3 where the relative signal intensity reflects the relative number of protein/peptide molecules got recruited to enamel tissue^21^ (Fig. 7 and S3 Videos). The representation of the surface signals as 3D plots further elucidates the signal intensity calibration where the z-coordinate is representing the pixel value, brightness of each pixel, of the 2D image (Fig. 7B and S4 Videos). Our data translate the previously-proven finding that the phosphorylated serine residues controls the binding capacity of the AEP proteome/peptidome to the HA crystals^43^. Still, the immobilization capacity of our designed hybrid constructs should be assessed as well to evaluate their immobilization compared to the native candidates.

In conclusion, customized proteins/peptides pellicles is a novel preventive approach with versatile mechanisms of actions. Integrating different functional domains of the prevalent salivary proteins/peptides showed synergistic effect by reducing biomass fouling and/or inducing biofilm dispersion and/or and triggering bacterial cell death. These data are valuable for bioengineering precision-guided enamel pellicles for translational caries prevention.

## Supplementary Information

Supplementary Information.
