# Supplementary material for: Bioinspired caries preventive strategy via customizable pellicles of saliva-derived protein/peptide constructs
Source: Sci Rep. 2021 Aug 20;11:17007. doi: 10.1038/s41598-021-96622-y (PMC8379205; doi:10.1038/s41598-021-96622-y)
Supplement: Supplementary file 1 — Supplementary Information. [file 41598_2021_96622_MOESM1_ESM.pptx]

## Slide 1
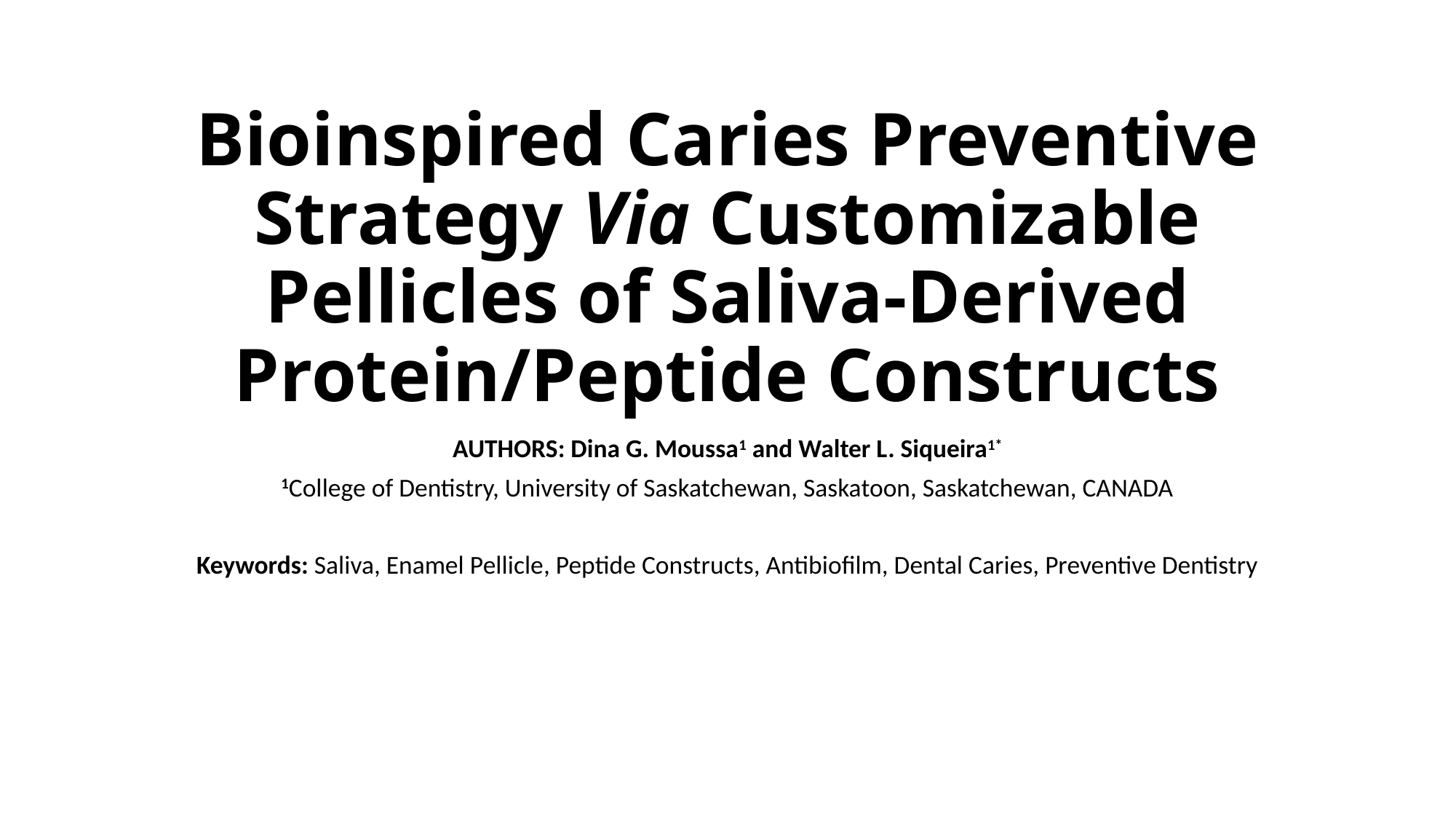

# Bioinspired Caries Preventive Strategy Via Customizable Pellicles of Saliva-Derived Protein/Peptide Constructs
AUTHORS: Dina G. Moussa1 and Walter L. Siqueira1*
1College of Dentistry, University of Saskatchewan, Saskatoon, Saskatchewan, CANADA
Keywords: Saliva, Enamel Pellicle, Peptide Constructs, Antibiofilm, Dental Caries, Preventive Dentistry

## Slide 2
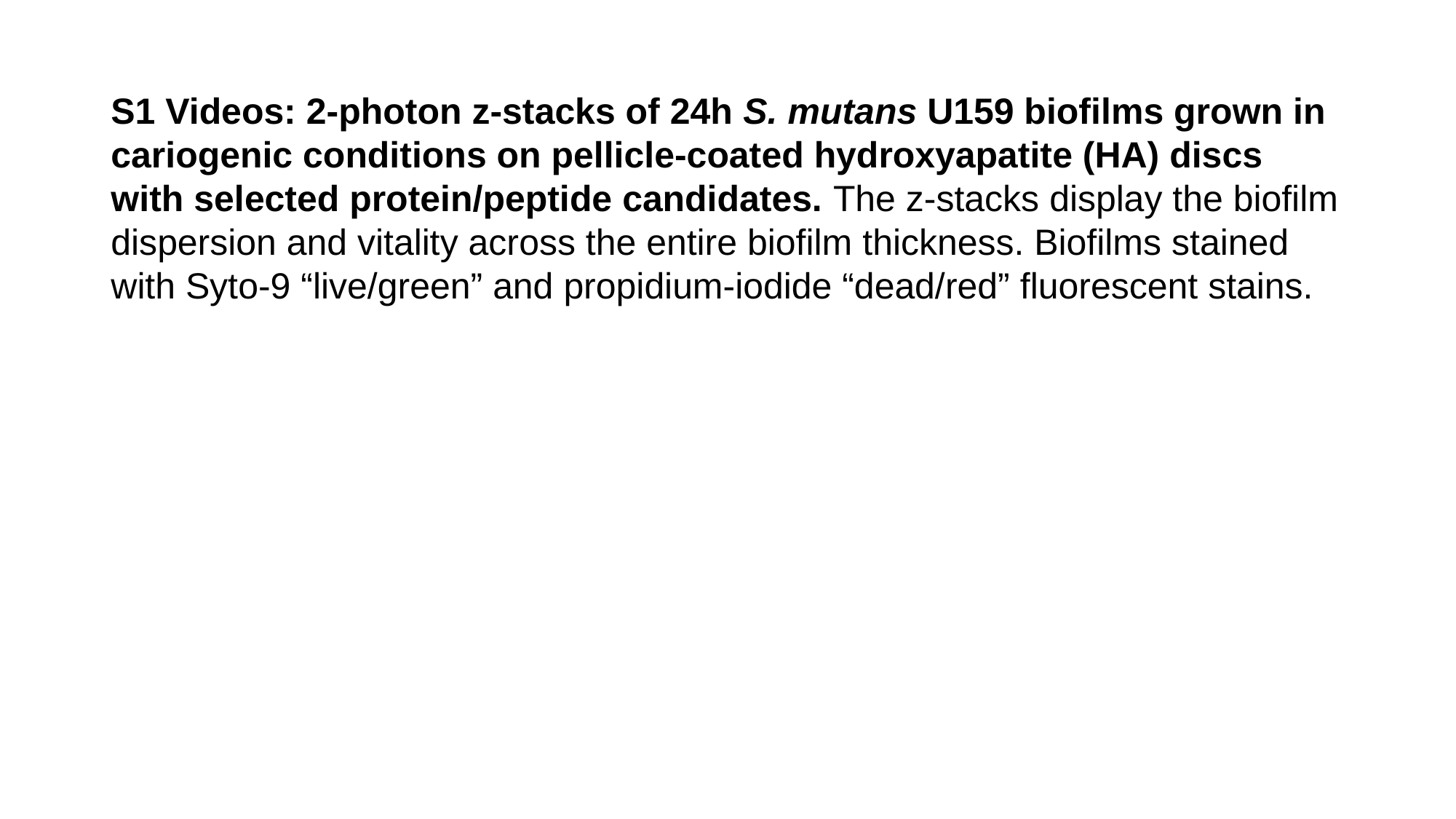

# S1 Videos: 2-photon z-stacks of 24h S. mutans U159 biofilms grown in cariogenic conditions on pellicle-coated hydroxyapatite (HA) discs with selected protein/peptide candidates. The z-stacks display the biofilm dispersion and vitality across the entire biofilm thickness. Biofilms stained with Syto-9 “live/green” and propidium-iodide “dead/red” fluorescent stains.

## Slide 3
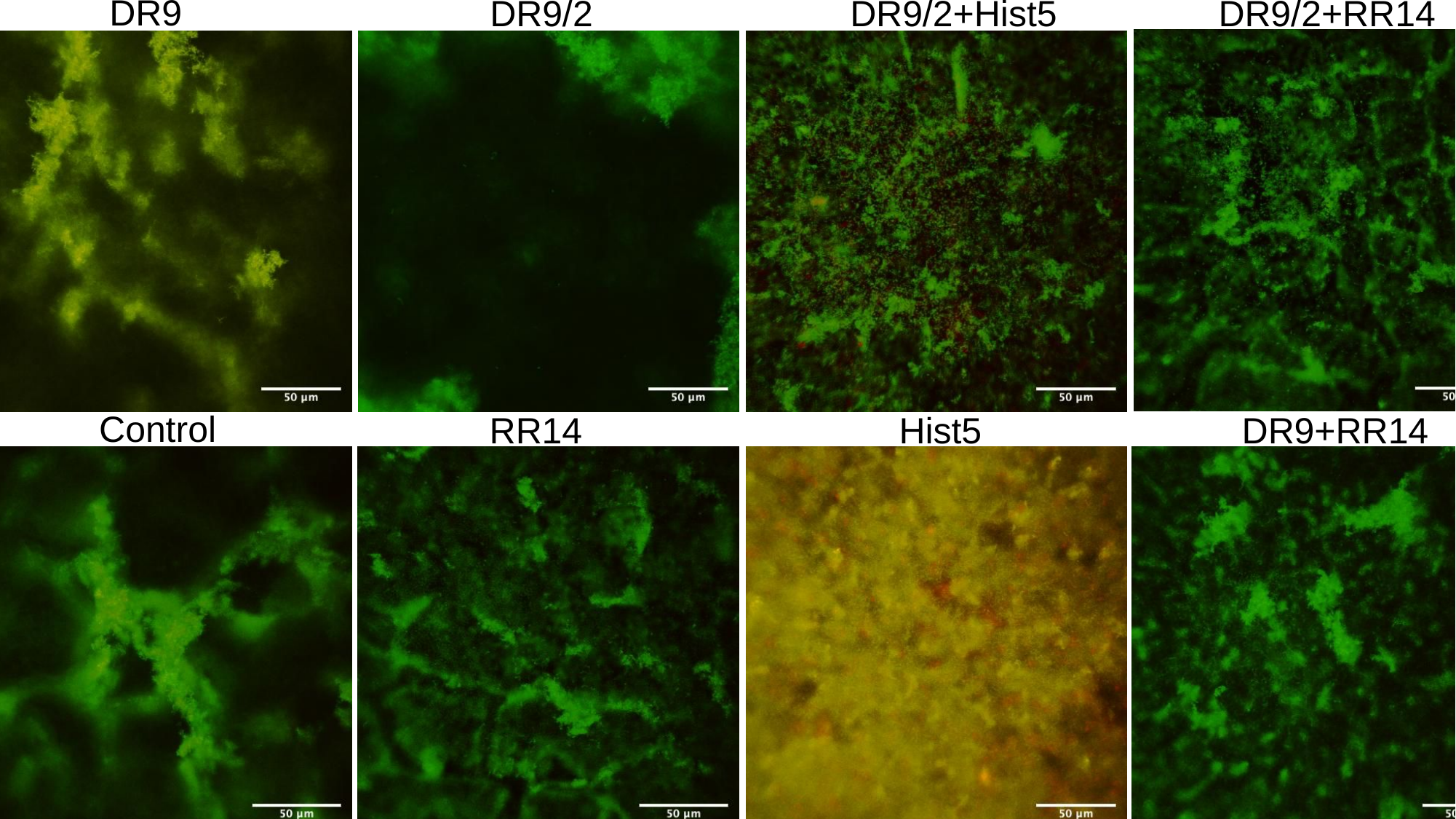

# DR9/2+RR14
DR9/2+Hist5
DR9
DR9/2
Control
RR14
DR9+RR14
Hist5

## Slide 4
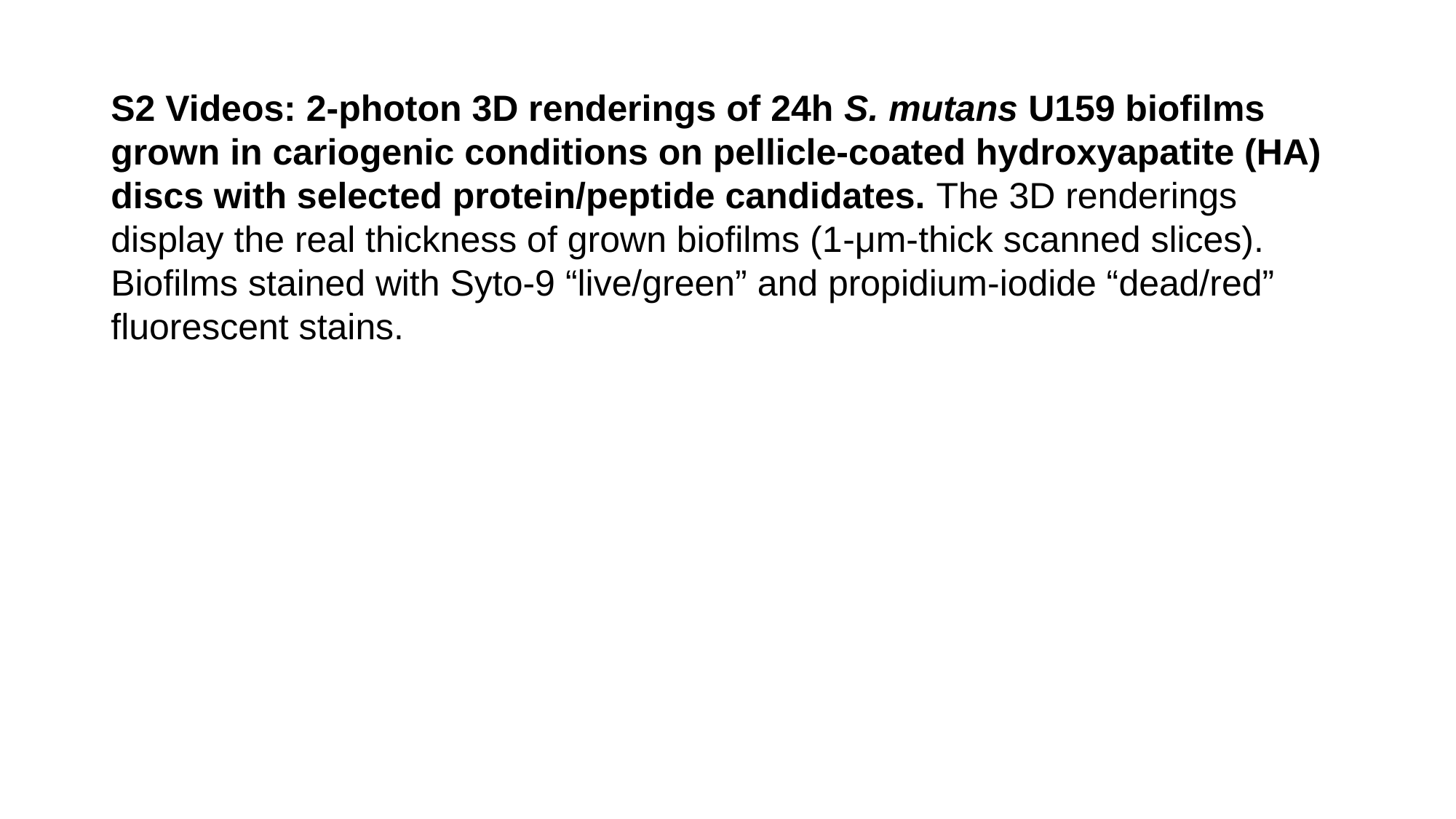

# S2 Videos: 2-photon 3D renderings of 24h S. mutans U159 biofilms grown in cariogenic conditions on pellicle-coated hydroxyapatite (HA) discs with selected protein/peptide candidates. The 3D renderings display the real thickness of grown biofilms (1-μm-thick scanned slices). Biofilms stained with Syto-9 “live/green” and propidium-iodide “dead/red” fluorescent stains.

## Slide 5
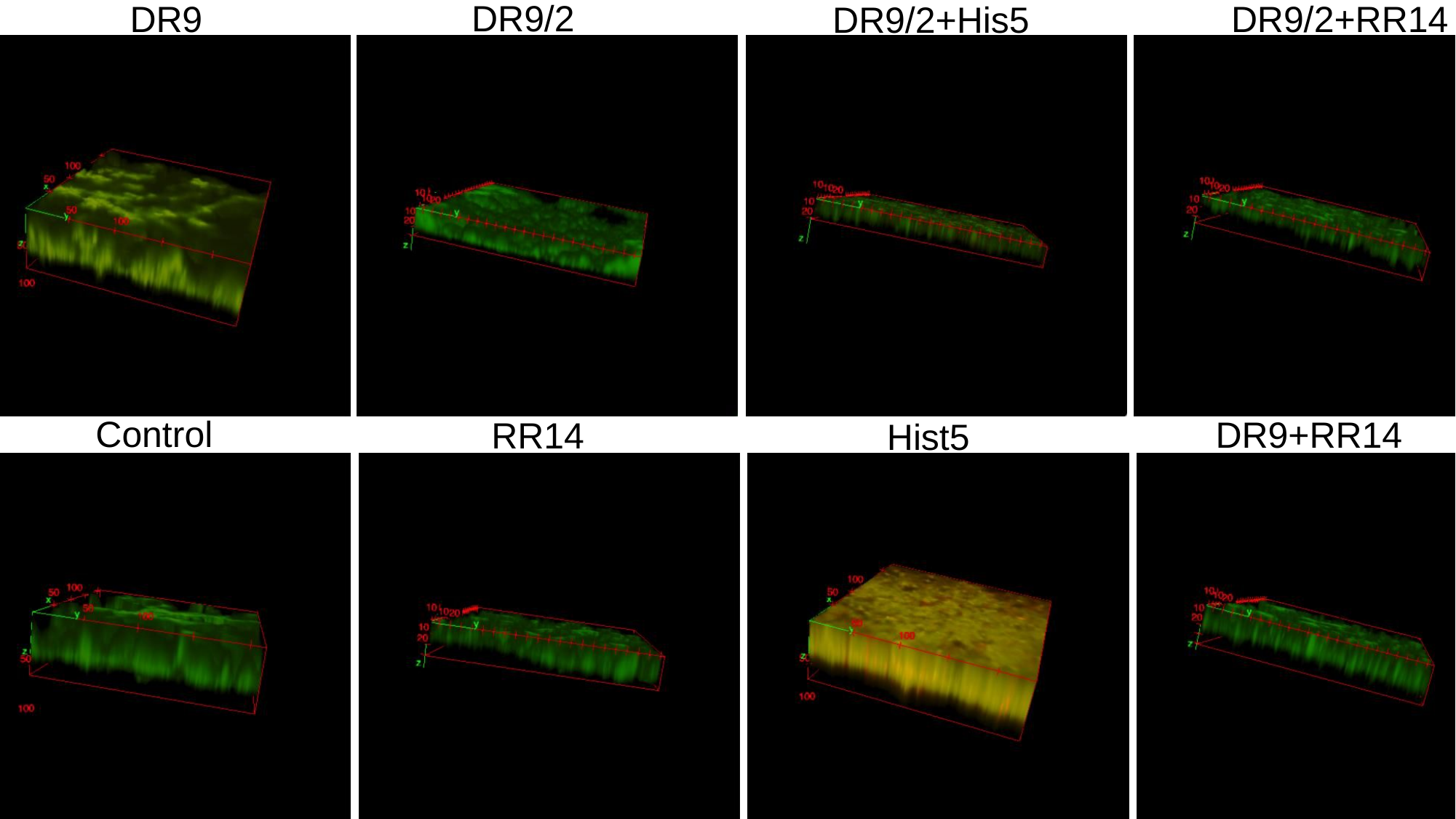

DR9/2+RR14
DR9/2
DR9
DR9/2+His5
Control
DR9+RR14
RR14
Hist5

## Slide 6
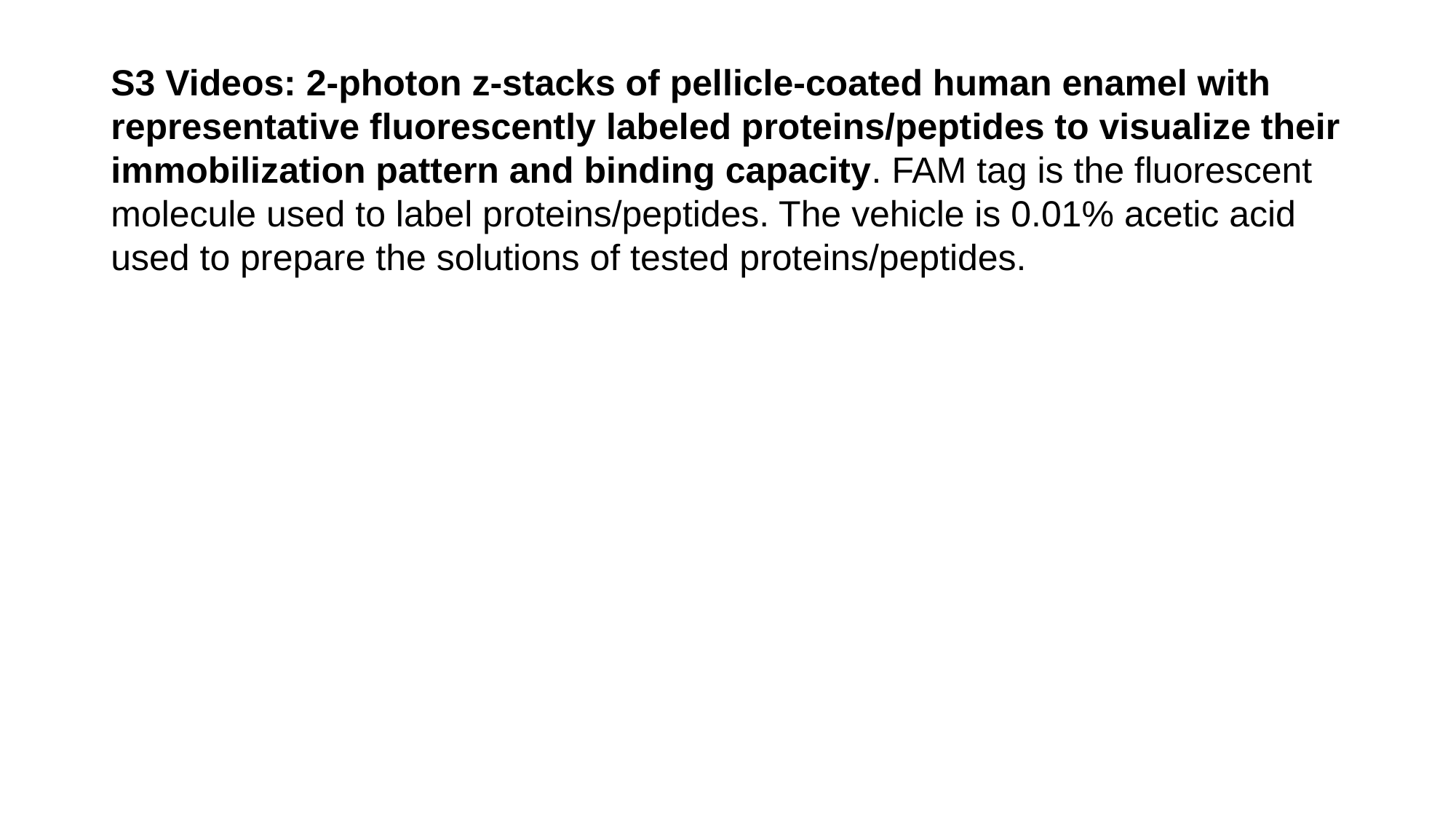

# S3 Videos: 2-photon z-stacks of pellicle-coated human enamel with representative fluorescently labeled proteins/peptides to visualize their immobilization pattern and binding capacity. FAM tag is the fluorescent molecule used to label proteins/peptides. The vehicle is 0.01% acetic acid used to prepare the solutions of tested proteins/peptides.

## Slide 7
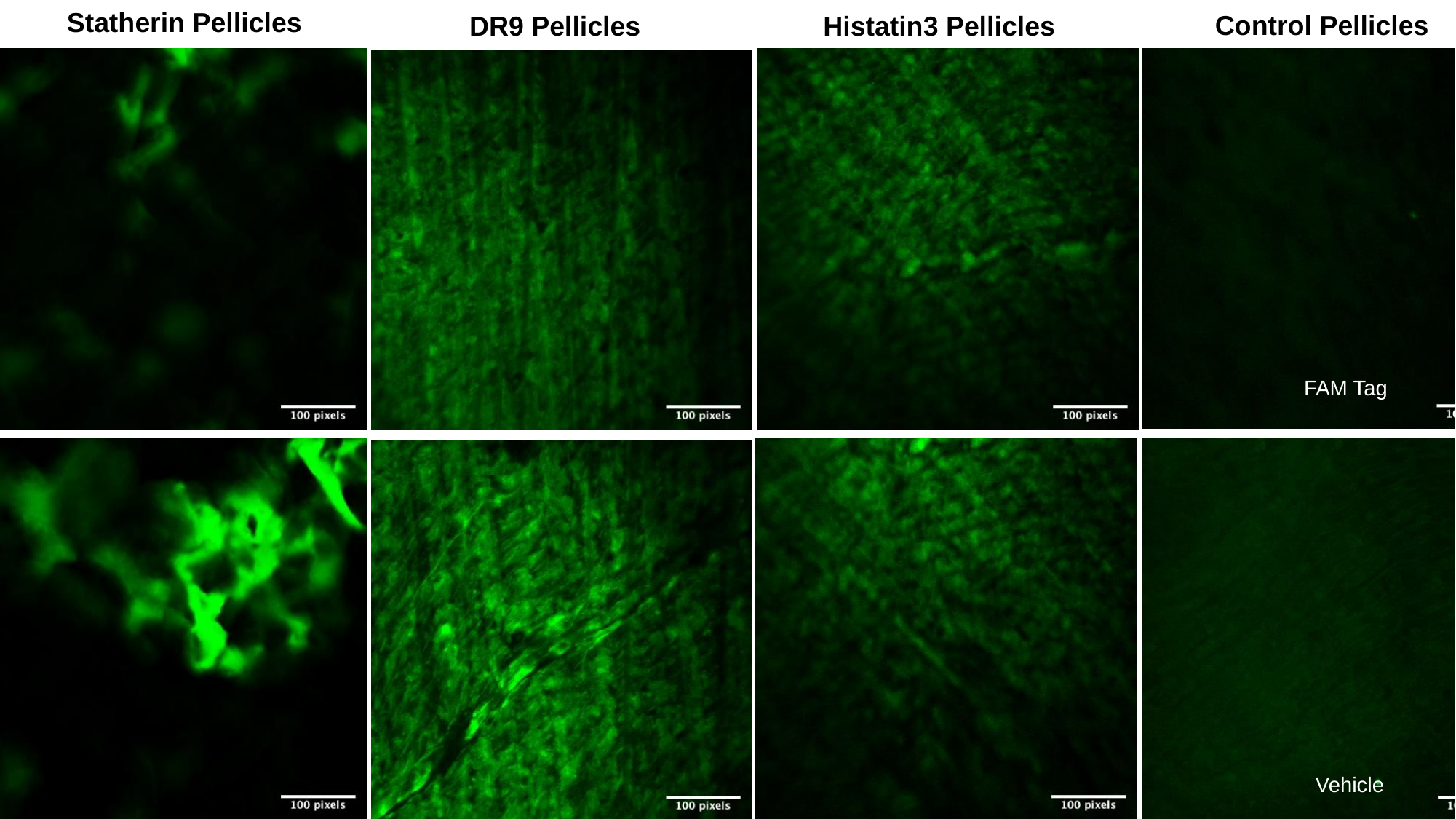

Statherin Pellicles
Control Pellicles
DR9 Pellicles
Histatin3 Pellicles
FAM Tag
Vehicle

## Slide 8
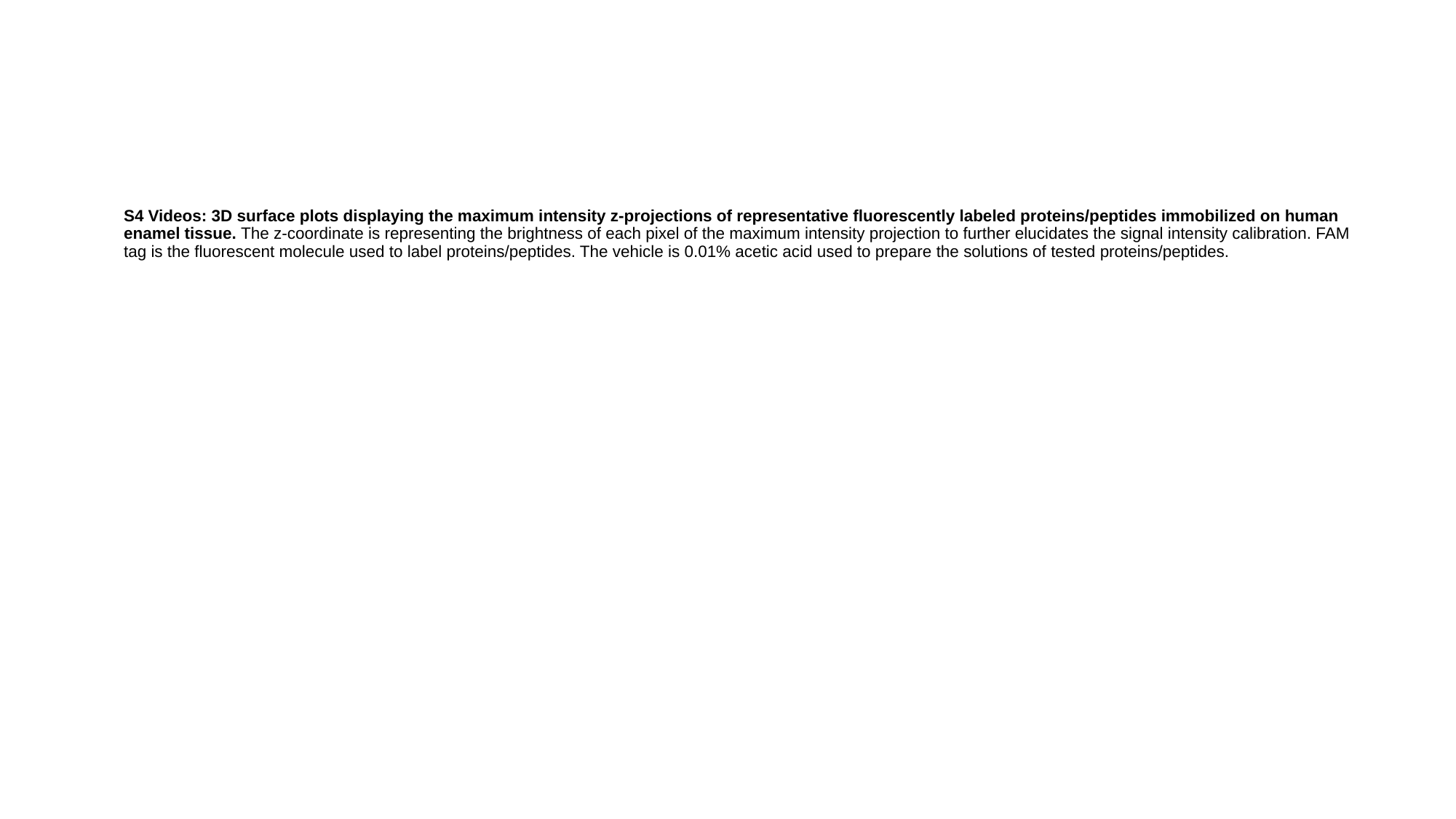

# S4 Videos: 3D surface plots displaying the maximum intensity z-projections of representative fluorescently labeled proteins/peptides immobilized on human enamel tissue. The z-coordinate is representing the brightness of each pixel of the maximum intensity projection to further elucidates the signal intensity calibration. FAM tag is the fluorescent molecule used to label proteins/peptides. The vehicle is 0.01% acetic acid used to prepare the solutions of tested proteins/peptides.

## Slide 9
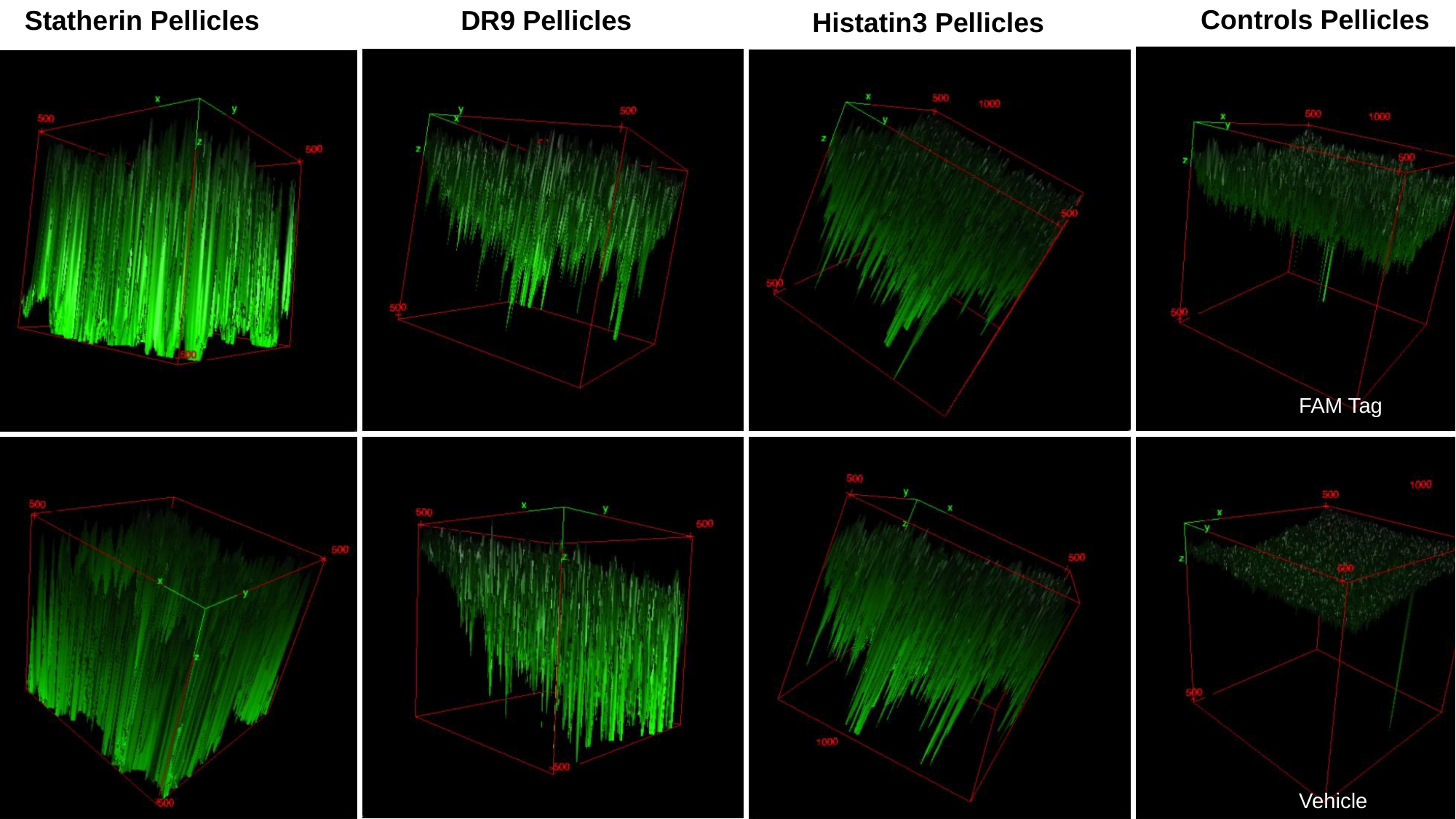

Histatin3 Pellicles
Controls Pellicles
Statherin Pellicles
DR9 Pellicles
FAM Tag
Vehicle
